
JOURNAL INFORMATION
==============================
NLM Title Abbreviation: Med Chir Rev
NLM Journal ID: 0034617

The Medico-Chirurgical Review

Publisher: Biomedical Journal Digitization Project

ARTICLE INFORMATION
==============================
PMCID: PMC4966177
PMID: 29917600
Article version: 1
Subjects: Articles

Varieties

Print publication date: 1828 Jul 1
Volume: 9
Issue: 17
First page: 169
Last page: 192
1828 Apr 26
License: This work is free of known copyright restrictions. For more information, please see PMC Back Issue Digitization.
License URL: https://creativecommons.org/publicdomain/mark/1.0/

==============================

25. Tubercular Affection of the Skin. By Dr. John Crampton.

[Transactions of the Association, vol. V.]

We have just received the fifth volume of the Dublin Transactions, and beg once more to congratulate our Irish brethren on the zeal and intelligence they continue to display in the work before us. This volume contains twenty papers, some of them of great length, and varying much, of course, in value. All these papers we shall examine in their turn; but, in the present fasciculus, we are obliged to select two or three of the shortest for the Periscope.

Dr. Crampton observes, that disorders of the skin are more or less connected with the state of the digestive and nutritive functions; and he is "disposed to think that still further insight may thus be obtained from external appearances on the surface, as to the condition of some of our most important inward organs, in point of soundness and integrity, or the reverse." But to the case.

J. Byrne, aged 56, a small emaciated man, was admitted into hospital for diarrhœa and rheumatism—but it was soon perceived that he laboured under phthisis, supposed to be tubercular. The appearance of the skin, however, attracted much attention.

"Large freckles and dark brown olive spots of various shape and size occupied the cuticle in patches; the interstitial spaces were only a shade lighter, being of a dirty dingy colour. In addition a number of tubercles of the same colour were observed on the trunk and arms, some broad, and gelatinous to the touch, about an inch long, and half an inch broad; others harder, like papulæ, about the size of a split pea; others again of a pyramidal form, attached to the surface by narrow, tough, peduncles. Some of these were about half an inch long, and about a quarter of an inch across their depending extremity. Those latter were hard to the touch, and felt cartilaginous, compared with the broad and boggy excrescences which were observed on some parts of the skin. But in those soft prominences small pisiform and hard nuclei were perceptible both to the sight and touch, as may be seen by inspecting the annexed drawing."

The patient stated that he had enjoyed good health till within twelve months of this time; but the alteration of the skin was of five years' duration, though it did not interfere with his health or avocations. He had been dirty and intemperate in his habits. The treatment was palliative, and the patient gradually sunk under the symptoms of phthisis. Dr. Crampton was anxious to ascertain if the tubercular diathesis should manifest itself in any of the inward organs or tissues. The following is the post-mortem examination.

"The body was wasted to the utmost degree of emaciation: the large freckles and irregular brown spots had disappeared after death, or were rather merged into a general dirty brown colour of a lighter shade, but of a uniform appearance. No change had taken place in the tumors or tubercles; the round and oval papular tubercles were of a caseous texture, penetrated the corion, and lay imbedded on the subjacent cellular tissue. On removing the cuticle a gelatinous substance appeared to pervade the cellular texture, especially under the broad and soft prominences; this gave the surface underneath a marbled appearance. The gelatinous substance afterwards disappeared, having become dissolved in the water, in which portions of the skin were macerated, and did not appear to have any connexion with the external marks on the skin. The pyriform tubercles did not penetrate deep into the corion; on cutting them out they were found to consist of a tough, white, hard, fibro-cartilaginous substance, no blood-vessels being visible through their texture. Some of the larger cartilaginous excrescences were hollow, and contained a glairy fluid.

"The lungs exhibited a diseased mass; tubercles in every stage. In the upper portion of the lobes the tubercles had degenerated into abscesses in a state of suppuration, and into cavities; in the middle portions tubercles beginning to soften, whilst in the lower they were small and hard. The heart was remarkably small, but the subject was rather diminutive. The liver hard, and dark-coloured, not enlarged, but giving a gritty feel to the knife; in fact throughout occupied with the *tubercula diffusa* of Dr. Farre. The spleen likewise in the same diffused tuberculated state. Mesenteric glands and kidneys free from disorder. The mucous membrane of the intestinal canal red, and attenuated, with slight ulcerated excoriations, such as are usually met with in phthisical patients."

Dr. C. thinks we must view the cutaneous disease, in this case, as a part of the general tubercular disposition, slowly but surely advancing—breaking up the texture of organs essential to life, and injuring the appearance and functions of those, at first sight, of less importance, but which, in the general harmony of the animal economy, cannot be overlooked. After remarking on the frequency of tuberculated conditions of various organs in other animals besides man, the Doctor informs us that—" not long since, when carving a hare at his own table, he was surprized to find the whole of the *fleshy and muscular* parts (what is the distinction between flesh and muscle?) of the body and limbs studded with white, cheesy, round tubercles, about the size of small peas." The Doctor adds—" my curiosity did not induce me to taste it." Dr. C. thinks we have no evidence of the removal of tubercles from the lungs—our object, therefore, must be, the prevention of their passing from the dormant into the active state. How is this best achieved?

"There are perhaps no measures which conduce more to prevent this unhappy termination, than a selection of good air, or a suitable climate, frequent change of scene, exercise in a carriage, riding, a long journey, or a sea-voyage. The diet should be nutritive, light, and restorative; the digestive organs, and the bowels especially, should be maintained in an active and healthy state. On the other hand, over-stimulating diet and fermented liquors in any thing more than what might be called minute portions, soon excite tubercular deposits, make them inflame, and undergo the successive changes already described, of which there was so excellent an illustration in the case before us."

---

### 26. CASE OF NÆVUS MATERNUS CURED BY VACCINATION.

[Glasgow Royal Infirmary.*]

Jane M'Gilpin, æt. 3 months, was admitted under the care of Dr. Young, October 22d, 1827. Has a roundish tumour on right side of chin, about the size of a sixpence, and prominent; it is of a purplish colour, and is apparently formed of blood-vessels. At birth, tumour was the size of a split pea, and has since been gradually increasing. Small punctures were made over surface of tumour, and vaccine lymph freely applied. In the course of eight or ten days, the tumour had become a vaccine pustule: a scab was thrown off, leaving the base of the tumour not quite eradicated; another suppuration took place, and a third, which, in the course of the ensuing month, effected a cure. Very important effects result from this simple method of treatment. Cow-pox is established in the system, whilst the tumour is as effectually removed as by the application of the ligature, or extirpation by the knife, (which are often productive of much constitutional irritation)—and, consequently, supersedes the necessity of these painful operations. Should the tumour be small, one puncture may suffice—if large, it will be necessary to vaccinate at several points. The size of the tumour is no objection to this method of treatment. The reporter is informed by Dr. Young, that he has attempted this practice in a case which occurred lately in private practice, and with the same beneficial results, although the cure was rather more protracted.

* Mr. Plymsoll's reports.

---

### 27. ON WHITLOW.

In the April number of our Edinburgh cotemporary, there is a very extended article (26 pages) on the subject of whitlow, in which Dr. Craigie has expended a prodigious quantity of medical and surgical erudition, in order to prove the good effects of early and deep incisions, when there is inflammation in the interior of the "digital synovial sheaths." In former times, there were numerous and able advocates for what Dr. Craigie and M. Roux call " premature incisions," in these deep-seated inflammations. In modern times, incisions (not premature) are advocated on a principle which Dr. Craigie contends to be erroneous—the giving issue to matter. " It is therefore said that the surgeon must wait until he detect fluctuation—and then only should he use the knife."

"This reasoning, (says Dr. Craigie,) which is founded on a loose analogy derived from the general rules of opening abscesses, is erroneous in the extreme. It is the delay till suppuration takes place that renders incisions of little or no avail. The interior of the digital synovial sheaths is by no means similar to a portion of cellular membrane. They are fitted for containing the flexor tendons; and the very circumstance which enables them to give these tendons support and resistance in the motions of the fingers, viz. their dense and unyielding character, prevents the sheaths from doing more than merely containing them. The moment inflammation commences in their cavity, as it spreads quickly over the whole, the swelling which ensues diminishes the cavity so much as not only not to contain easily the tendons there placed, but to induce quickly an extreme degree of compression. The unyielding nature of the fibrous case causes it to operate like a tight ligature over the whole course of the tendons. It thereby arrests mechanically the circulation within them; and this, as in other textures, speedily terminates in death of the tendon."

"For these evils the natural remedy, I conceive, is much earlier employment of the incision. To be efficient, indeed, the principle of this measure requires to be completely changed. Instead of using it with the view of opening an abscess and discharging purulent matter, it should be employed, first, as powerful and efficient means of local depletion; secondly, as the means of preventing that compression which, by its mechanical power only, must speedily arrest the circulation in the tendons, and thereby produce mortification of their texture."

We apprehend that Dr. Craigie is mistaken in supposing that modern surgeons are in the habit generally of delaying the incisions in deep-seated whitlow, till there is evidence of matter being formed. It cannot be doubted that such a man as M. Roux should be acquainted with the sentiments and practice of the surgical world on this point. Let us hear what he says on the occasion.

"Ces incisions prématurées, faites pendant la durée de la periode inflammatoire du Panaris, se trouvent recommandées par un assez grand nombre d'auteurs anciens, qui, a leur faveur, croyaient pouvoir donner issue à la sérosité âcre et corrosive qu'ils pensaient être cause de la maladie dont il s'agit ici:—*elles sont encore mises en pratique, par la plupart des chirurgiens modernes, dans le double but de faire cesses la douleur, et de s'opposer, en arrêtant le progres du panaris, &c.*"*

Here, then, we find Dr. Craigie expending immense time, labour, and ink, to prove the propriety of the practice of "the majority of modern surgeons." It is not a little curious, that the very surgeon from whom we have quoted the above passage, is labouring hard to shew that this modern-ancient practice is decidedly injurious, and ought to be discontinued!

M. Roux divides whitlow into four species, according to the seats of the inflammation. The first is seated in the skin of the finger, and is merely an erysipelatous whitlow—the second affects the cellular tissue between the skin and the sheaths of the flexor tendons—denominated by M. Roux phlegmonous whitlow. The intensity of this species seems to depend on the density of the inflamed tissue, the great number of nervous filaments distributed through it, and the paucity of extensibility in the skin which covers it. The third species is, without doubt, the most serious of all. It attacks the sheaths of the tendons and their synovial membrane, whence it sometimes propagates itself to the phalangeal articulations. It is in this species of whitlow that we most frequently see collections of matter form in the hand, fore-arm, and other parts of the upper extremity. The fourth species is an inflammation of the periosteum, in which the phalanges are often stricken with necrosis, and the finger lost for all useful purposes. The second and third species of whitlow are often combined—or the second spreads and causes the third. It is not, therefore, an easy task always to discriminate between them. The advocates, says M. Roux, of early incisions tell us that these incisions, when freely made into the second species of panaris, will prevent the

* Dictionnaire de Medecine, Vol. XVI. p. 136. 1827.

extension of the inflammation to the sheaths of the muscles. This, M. Roux observes, is contrary to the most positive facts—"contre ce que demontrent chaque jour les faits les plus positifs." In the second, or phlegmonous species, he says, the presence of matter is sometimes difficult to be ascertained—and, in the third species, it requires all the discrimination and tactus eruditus of the surgeon to recognise the stage of suppuration. This eminent surgeon maintains that it is of the greatest consequence to distinguish between the stage of inflammation and that of suppuration, as the treatment ought to be totally different. In the first, or inflammatory stage, he recommends the most energetic local antiphlogistics. At the very commencement of deep-seated whitlow, he has found the greatest advantage from the application of numerous leeches, not to the immediate seat of the inflammation, but around the root of the finger. After these local bleedings, repeated according to circumstances, he employs emollient fomentations and poultices, mixed with opium which gives great relief from pain.

M. Roux next discusses the question of early incisions. He acknowledges that he has the majority of his surgical brethren against him—that the anatomy of the parts inflamed in whitlow gives a most specious plausibility to the doctrine of his antagonists—but facts are, nevertheless, opposed to theory. "La theorie parait ici des plus satisfaisantes; et cependant les faits pratiques, ecueil de toute explication hypothetique et hasardée, *dementent tous les jours les heureux effets des incisions prematurés.*" The following are the results of M. Roux's observations and experience.

"An incision made before the formation of matter appears to diminish the pain and weaken the intensity of the whitlow, when the inflammation is confined to the sub-cutaneous cellular tissue; but in this species of panaris the pain is never very urgent, and therefore requires not the use of these incisions. But are we to make these incisions, as many advise, to prevent the extension of inflammation to the tendinous sheaths beneath? This measure has never appeared to me to attain the object in view. I have invariably seen, notwithstanding the section of the skin and subcutaneous tissue, the pain and other symptoms persist—the suppuration become established in the sheaths of the tendons; while other and numerous facts have convinced me that the inflammation of phlegmonous whitlow does not spread to the said sheaths, except where the incisions have been made. In this species, (phlegmonous,) should we penetrate the sheaths of the tendons, or carry our incisions down to the bone, as many of our *patricians* advise? The surgeon will probably feel regret at having mistaken (a thing very easily done) a subcutaneous inflammation for inflammation of the tendinous sheath, and thus unnecessarily laid bare the tendons of the finger. I have no hesitation, therefore, in rejecting entirely these early incisions, as most frequently useless, and sometimes dangerous."

In the second, or suppurative stage, M. Roux is an advocate for a free opening, carried to the seat of the suppuration, whether deep or superficial.

We have thus set the sentiments of two authorities, Dr. Craigie and M. Roux, before our readers, leaving it for them to determine which they are to take for their guide. It is sufficiently evident that Dr. Craigie's treatment, by early incisions, is not only old, but the general practice of the surgical world—at the present time. M. Roux, therefore, has the more difficult task—that of persuading surgeons to change their adopted plans, while Dr. Craigie's labour seems almost a work of supererogation—being the recommendation of that which is already general.

---

### 28. Hydrocele cured by "Astringent Applications."

In the Révue Médicale for February, M. Mannoury has made some observations upon Hydrocele, and detailed a case successfully treated without an operation.

M. M.——, æt. 62, of good constitution, found the left side of his scrotum gradually increasing in size for three or four years, at the end of which time he applied to our author, who readily recognized a common hydrocele. Six months afterwards, the tumour was as large as one's fist, and as the patient had not sufficient resolution to submit to an operation, he was directed to macerate a quantity of oak-bark in some forge-water, to add a little vinegar, and employ the whole as a lotion to the scrotum twice a day. It was continued during the Summer, and at the expiration of a year, when M. Mannoury saw the patient, the hydrocele had entirely disappeared, and nothing but a slight enlargement of the testicle remained.

This mode of treatment is applicable only to small collections of fluid in the tunica vaginalis, but we think it is worthy a trial in weakly patients, and especially old persons, who sometimes have not courage to undergo the operation, or stamina afterwards to support its effects. We remember, not very long ago, seeing the water evacuated by means of the caustic potash; a severe attack of erysipelas followed, which put the old man's life in considerable jeopardy; indeed, if we are not mistaken, proved fatal. Of course, where the patient is occupied, as in business; or, in fact, wherever the common operation is advisable, this plan of treatment is inadmissible, because it is both tedious and uncertain.

---

### 29. CASE OF EXTREME DIFFICULTY OF BREATHING CURED BY THE HYDROCYANIC ACID. By MICHAEL RYAN, M.D.

[Dublin Transactions, Vol. V.]

A young married woman, aged 23 years, and nursing at the time, retired to rest in perfect health, on the night of the 25th September. She had occasion, in the course of the night, to get up, and expose herself to the chilling influence of the night air at the door. She was almost immediately seized with pain in the chest, and extreme difficulty of breathing, threatening suffocation. Early next morning she was bled to eight ounces, without relief. At 2 p. m. our author found her sitting in bed, breathing most laboriously—the inspirations long—expirations short—face pale—lips livid—features relaxed—eyes glassy—no pulse at the wrist—action of the heart feeble and indistinct—hands and feet covered with a clammy perspiration.

As it was evident that the lancet could not be used, on account of the state of the circulation, and as it appeared probable that she would die before a blister could rise, Dr. R. determined to try the prussic acid, having repeatedly found it the most efficacious remedy in relieving difficult respiration and oppressed circulation.

"Accordingly six minims of the acid, as prepared by Scheele, were mixed in two ounces of distilled water, with one drachm of compound spirit of lavender. Of this mixture she was to take half an ounce every ten minutes, until her breathing was in some manner affected. I also directed that if any relief were obtained by the first, second, or third dose, the medicine should not be repeated oftener than every half hour, and that it should be discontinued altogether, and the administration of a little brandy substituted if sudden weakness, headach, or fainting should supervene.

"So hopeless was this case, in my opinion, that I thought it most probable that the woman would be dead before the medicine could be administered.

"Four o'clock, p. m. The medicine has been taken as directed; the breathing is much easier, and the patient is altogether wonderfully relieved. She complains of some debility. Six o'clock, p. m. Is not so well as at last report; complains much more of weakness. Let her have some brandy in small quantities during the next hour. Seven o'clock, p. m. Pulse very weak, small, and frequent. Says she experienced the greatest relief from the mixture, which is all taken; but the shortness of the breathing has been increasing for the last two hours, and is now as violent as at two o'clock, p. m. Her countenance expresses extreme anxiety. On the whole her case seems perfectly hopeless. Let her have eight minims of the acid with two grains of tartar emetic in two ounces of distilled water, half an ounce to be taken every ten minutes as above directed."

By the time she had taken four doses of the mixture, considerable ease was obtained—and after the last dose she fell into a sleep, from which she awoke free from all alarming symptoms. The mixture acted on her stomach and bowels. In this case 14 drops of hydrocyanic acid were exhibited in the space of eight hours. The acid was obtained from Messrs. Stanley, and Co., and was prepared according to the formula of Scheele.

The Doctor remarks that this remedy is extremely dangerous, and requires the closest observation of the practitioner. The most powerful stimulants should be immediately had recourse to, when its deleterious effects on the constitution are perceptible.

---

### 30. BILIARY SECRETION.

Mr. Abernethy, in his 35th Lecture, takes up some pages with the consideration of biliary secretion. He says, "the function of the liver is a strange thing—it is what does not take place in any other part of the body." We do not see any thing very strange in this, as there are no livers in "any other parts of the body." But the strangeness to which he alludes is the secretion by a *vein*. But is there nothing particular in the vena portæ? It is like no other vein. All other vessels of this kind arise from the terminations of arteries—progressively enlarge—and end in the heart. This is not the case with the vena portæ. It arises from the terminations of arteries, it is true; but it does not progressively enlarge, and end in the heart by a large trunk or trunks. No. After its branches have enlarged, and formed one great trunk, this trunk splits into a great many branches which progressively decrease in calibre, and end by innumerable capillaries in a gland. Where, then, is the analogy between the vena portæ and other veins? From the moment that this vessel begins to subdivide and push its innumerable ramifications into the substance of the liver, it assumes the great anatomical character of an artery—progressive decrease of calibre. Where is the wonder, then, that it should take on the function of an artery? If the cavæ hepaticæ indeed, which shew the venous character in shape and structure, were to secrete bile, we should say it was "a strange thing."

Mr. A. clings to the glory of a discovery in having found a lusus naturæ, where the vena portæ terminated in the inferior cava—where the *hepatic artery was one-third larger than common*—where the gall-bladder was *one-third smaller* than usual—where there was some little, *very little* bile found in it—and, in short, where the child had lived a year after birth. Now, in all this, we cannot see any thing to "completely quash" the ancient and the modern opinion that there is something peculiar in the vena portæ, as compared with other veins, for the purpose of biliary secretion. It is but rarely that a lusus naturæ proves any thing in physiology. Nature had here enlarged the hepatic artery, in order to carry blood to the liver in place of the vena portæ—but, in all fairness, this does not prove that the *latter* vessel is not more proper for biliary secretion than the FORMER. A great deal too much importance has been attached to this lusus, both by Mr. Abernethy and others, who do not appear to have taken into consideration the anatomical difference between this vein and the other veins in the body. For our own parts, we are not ashamed to subscribe to the old opinion, that the vena portæ is the vessel that supplies the pabulum of the biliary secretion in the liver—and that the hepatic artery (in all cases except the lusus naturæ in question) is the nutrient vessel of the organ.

---

### 31. DISSECTION WOUND—DEEP INCISIONS.

Dr. Evans has recently published a case which occurred in the Malin Dispensary, and where the practice of deep incisions appears to have been attended with most beneficial consequences.

The patient, Mr. Layrd, a surgeon, while opening an abscess in the scrotum of a man, whose urethra had sloughed, received some morbific matter in consequence of being scratched with the point of a pin. Acute pain was immediately felt—and in the same evening a train of severe complaints, now too well known to need detail, commenced. The patient's sufferings are said to be indescribable by language. The pain and burning sensation were confined at first to one spot—where the pin had entered. He ran from place to place, unable to support the torment. He scarified the part—applied caustic—put the hand in hot water, but with aggravation of his sufferings. Cold and other applications were equally inefficacious. Next morning, a crucial incision was made over the thumb, and he was bled to 16 ounces, when he became faint. Calomel and salts produced free evacuations. Dr. Evans saw the patient 36 hours after the reception of the poison. He was tossing to and fro—face flushed—was gnashing his teeth—beating the bed with his feet—and crying out on account of the fiery sensation in the thumb, which was red and swelled. The lymphatics were visible, running up the arm to near the axilla. He was now obliged to keep his hand constantly in cold water often renewed. Three large leeches were applied to the wounded part, as also belladonna and hyosciamus in watery solution. "On the sound skin, above the termination of the inflamed lymphatics, an eschar was formed circularly, by pencilling the part with lunar caustic." Five grains of Dover's powder and one of quinine every second hour. On the third day, gentle pressure caused a discharge of dark fluid blood from the part. The pain, &c. were unabated. The part was freely incised till the scalpel reached the bone. Rollers were firmly applied to the thumb, hand, and arm, with decided relief. These were moistened with narcotic solutions. Leeches having been procured from a distance, the rollers were taken off, and 22 of these animals were applied over the head (hand no doubt) and fore-arm. No relief followed. More incisions were made, and dark grumous blood continued to ooze out. The pain, swelling, and inflammation continued to spread. On the 5th day, the constitutional symptoms being severe, and the arm swelled, a five-inch incision was made, and the tense fascia freely divided. Opiates, quinine, wine, and other remedies, were employed. Great ease immediately followed this incision; but the pain now invaded the fore-finger—and a deep incision here produced quick relief. His countenance looked haggard—features sharp—is restless and peevish—urine pale and passed in large quantities. He took animal food and drink freely. Some curious nervous symptoms now shewed themselves. In a consultation, it was determined to pursue the plan of incisions according to circumstances. Nourishment, wine, laudanum in pretty full doses. Several more incisions were made; but still, on the 9th day, we find "every symptom aggravated," and "he appears rapidly sinking, as well from disease as from diarrhœa." The superacetate of lead and opium prescribed. On the 10th and 11th days, the arm was frightful—phlyctenæ scattered over it. He took a bottle of Madeira and some malt liquor, with quinine, &c. daily. On the 12th day, the inflammation covered the shoulder—the diarrhœa had ceased—the quinine was given in doses of two grains every two hours. He takes two bottles of wine, besides malt liquor in the 24 hours—refuses medicine. On the 18th day, the arm was the size of a pillow, and fluctuation was evident throughout this extremity. From an incision near the origin of the supinator radii longus, a large quantity of healthy pus flowed out. Some other incisions were made, and the greatest relief was the consequence. Matter, on the 19th day, was evident on the side of the chest, and was discharged by an incision. From this time, the unfortunate patient gradually improved—and, on the 55th day, he was able to crawl out of doors for a few minutes. In the following sentiment we entirely agree—indeed it is the doctrine which we have always maintained.

"It is superfluous to refer to the many valuable cases published on similar affections. That now detailed justifies the opinion that neither any bad habit of body, or unwholesome condition of atmosphere, or the injury itself, could have availed any thing in inducing a disease so severe; and that all was caused by the minutest portion of an animal poison entering the system, and this poison generated during the life of the individual who communicated it. Similar occurrences do not often happen. Let the nature of the poison be what it may, it derived its virulence from extravasated urine, causing gangrene of the parts in contact with it, and involving extensively the cellular membrane. That the person imparting this poison was free from any disease till attacked with the retention of urine, may be inferred from his general healthy condition and his country life."*

We agree also with the able author, that venesection and other depletive measures had little or no good effect in this case. How can it be expected that they should? A poison is received—the nervous system is irritated—the fluids are

* Ed. Journal for April, 1828, p. 287.

contaminated—and depletion can only go to the relief—(a precarious relief)—of some of the inflammatory affections set up. The constitution must be supported till it can overcome the effects of the poison. The local incisions—even anterior to suppuration—were, no doubt, of great benefit.

### 32. State of the Perspiration in Acute Rheumatism.

Dr. Chambers' practice in this disease is already known to our readers, from some notice which we took of cases published in our cotemporary, the Medical and Physical Journal. The same distinguished physician has lately detailed two cases in the Medical Gazette, which were quickly cured by ten grains of calomel with two of opium at night, and a black draught in the morning. This process was preceded by one small bleeding. In the second case, a young man had acute rheumatism of the fibrous structures about the hands, wrists, fore-arms, &c. shifting its seat frequently, and attended with "*profuse acid perspiration.*" The pulse was 120—palpitation of the heart—pain in the cardiac region much aggravated by motion or any excitement. He was bled and put on the above plan. The pain continuing about the heart, he was bled a second time, and on the fifth day his mouth was sore from the calomel. The symptoms were diminished, though not removed. The calomel and opium, in smaller doses, were continued. A return of the palpitation required another bleeding, succeeded by a blister, when the disease yielded.

We have noticed this case, in order to draw the attention of our readers to the sensible properties of the perspiration in acute rheumatism. Dr. Chambers incidentally notices the acid quality of the cutaneous discharge in this case, and we should be glad to learn whether he has *generally* noticed such chemical property in rheumatic perspiration. We were lately informed by a medical gentleman, (Mr. Wigan,) that he has almost invariably ascertained the acidity of sweat issuing from the bodies of patients labouring under this disease. He employs alkalies very freely in acute rheumatism, and, he says, with decided benefit. He was led to this practice from finding a popular remedy for the disease, to consist principally of solution of the ashes of certain plants. We think we have seen the remark before, respecting this acidity of the sweat in rheumatism—and certainly we have often remarked the sour smell emitted from the patient's body; but we think it would be worth while to test the fluid in these cases, in order to put the matter beyond dispute.

### 33. Fracture of the Trochanters, followed by Tetanus.

In a weekly Journal, there is rather an extraordinary case related by Scotus, and certainly, if the particulars be correct, there is culpability attachable somewhere. It appears that an old man fell in the street, and was admitted, some days afterwards, into the Royal Infirmary, with the limb shortened "certainly two inches; the toes inverted, and the knee drawn above and across that of the opposite side." There was, at the same time, mobility of the limb, and from this circumstance it was determined, in consultation, that the case was not one of luxation, but fracture through the trochanters, and the long splint ordered accordingly. The patient seems, however, to have fallen into bad hands, for the reporter tells us that "the skin was completely torn off the instep by the process of extension," and there was produced, to boot, "a gash of an inch in depth, extending across the lower edge of the gluteus muscle, round into the groin!" Chronic tetanus came on, and was somewhat relieved by stimulants and a full diet, but the nates sloughed, and the patient sank. On dissection, there was found fracture of the trochanter major, kept together by the "thickened capsular membrane," with a considerable ossific deposit around the neck of the femur, and some absorption of the cartilage of the head of the bone.

This account of the dissection, and the statement respecting the *inversion* of the limb, are taken from Dr. Hunter's clinical lecture on the case; but Scotus asserts, that the limb, on removing the bandages, was *everted*, and the neck of the femur almost entirely removed by interstitial absorption. With such contradictory statements before one, it is exceedingly difficult to know what to say to them; but it seems to us, that that peculiar affection of the neck of the femur, viz. an interstitial absorption, which we meet with in old persons, had taken place, or was taking place, here, *prior* to the occurrence of the fracture across the trochanter. Scotus makes some remarks upon the application of the long splint, the drift of which, as far as we can comprehend them, is to condemn its application. We must say, however, that from what we have seen, the long splint is of very great service indeed in fractures of the thigh, and we should be induced to prefer it, in the generality of cases, to the double inclined plane, or any other method. The *manner* in which it was applied in this particular case we do not mean to defend; in fact, we must say, that the dresser whose bandages can produce gashes *an inch in depth*, from the glutæus muscle to the groin, deserves to have a few "gashes" inflicted on his own glutæus by the cat o'nine tails. We are not to be understood as making any reflexions upon the surgeon, Dr. Hunter; for we dare say the statements of Scotus are a little touched up and coloured, as has *occasionally* happened with the productions of his brother reporters on this side the Tweed.*

---

34. Mr. Gaitskell's Obstetrical Bandage, or Bandage of Support.

This bandage is applicable to four different periods of parturition.

1*st*. *The Eighth Month of Pregnancy.* At this period, the abdomen is often pendulous, particularly in fat women, and those who have borne many children. The over-stretching of the abdominal muscles destroys their tone, and lessens the elasticity of the integuments, which produce pain in the lumbar region, and many uncomfortable feelings. These are greatly relieved by the application of the bandage, which should be placed under the linen, and tied in the middle of the loins.

2*dly*. *At the Commencement of Labour.* In this instance, the bandage should be applied exterior to the clothes, and tied on the right side of the abdomen, the patient lying on her left. It will be applied with more facility in the erect position of the trunk.

The pressure must be regulated by the feelings of the patient, as the integuments and fascia are in some cases exquisitely tender.

When the membranes are broken, and the waters discharged, the second row of tapes must be tied; by these means, the parietes of the abdomen are brought into contact with the enlarged uterus, which, embracing it, furnishes several additional points of support; this enables that organ to act with more energy in propelling and expelling the fœtus.

3*dly*. *After the Fœtus is expelled.* The third row of tapes must now be employed to lessen the abdominal cavity, and compress the uterus. At this period it is most essentially useful, by facilitating the action of the uterus in detaching and expelling the placenta.

4*thly*. *After the Expulsion of the Placenta.* Many a woman, after an easy labour, and early expulsion of the placenta, is subject to an atonic state of the uterus, followed by internal flooding and death, though there is no external appearance of hæmorrhage.

"In this way I know five instances of sudden death, the os tincæ closely contracted and the cavity of the uterus full of fluid and coagulated blood, and was not suspected till discovered by post-mortem examination. The proper application of the bandage completely prevents this afflicting misfortune.

"Another good effect is that of restoring the energy of the abdominal muscles and improving the shape.

"In illustration of the dangers pending on those females who are so unfortunate as to be delivered without a supporting-bandage, I subjoin a few cases."

A lady, aged thirty, of a delicate constitution, was brought to bed of a fine healthy child; had an easy labour, and the placenta followed in about 15 minutes, with no more than the usual discharge. As the patient felt a little refreshed, the accoucheur went down to his breakfast, but had scarcely begun, when

---

* Dr. Ballingall's account of the case will be seen a little farther on in this Number.—*Ed.*

the nurse ran down, and, in a flurry, stated her mistress was fainting; the accoucheur immediately visited his patient, and found her as described by the nurse, the face and skin pallid—the extremities cold—the pulse feeble, quick, and scarcely perceptible, while the abdomen was greatly enlarged. On examining the napkins, he found them unsoiled, and, on examining the vagina, the os tincæ was found closely contracted; upon pressing it with the finger, it produced pain, when much fluid and coagulated blood were expelled. He now thought it necessary to dilate the os tincæ, introduce the hand, and empty the uterus of its contents; and at the same time to give support, by pinning a napkin tight round the waist. By these means, the uterine contraction was completed, the hemorrhage stopped, and the patient finally, but with great difficulty, recovered.

The quantity of blood lost on this occasion, was calculated at more than two quarts.

A similar case occurred to the same gentleman a few years after, which induced him to employ a table-napkin as a bandage of support, on the completion of delivery; since which, in 40 years' extensive practice, he had the good fortune to meet with no more such distressing occurrences.

*Case* 2. A lady in this parish was delivered by a female midwife:—The labour was fair and easy, and every thing went on to her wishes; the midwife left her without pressing on the abdomen, or putting on a bandage, while she dedicated her time to the child. A few minutes after this, her patient grew faint, turned pale, and quickly expired. There was no external hemorrhage.

On the post-mortem examination, the uterus was found filled with fluid and coagulated blood, and extended to the scrobiculus cordis.

"The remarks I have to offer on these cases are, the necessity of compression of the abdomen immediately after the expulsion of the placenta, the examination of the pulse and countenance, and not too hasty a departure of the practitioner, are incumbent duties, and necessary to the safety of the patient.

"In all those who have been rescued, it has been accomplished by the prompt introduction of the hand into the cavity of the uterus for the discharge of its contents, aided by external compression, and the mechanical stimulus of the hand, bringing on its proper contraction. Should these fail, there is only one more resource, the transfusion of human blood, recommended by my friend Dr. Blundell.

"Both Dr. Blundell and Dr. Conquest have seen the bandage, and speak highly in favor of its utility."

We are informed by Mr. Gaitskell, whose experience has been almost unlimited, that, out of 7000 cases of midwifery, occurring in his practice during the last 45 years, he cannot recollect a single instance of fatal uterine hemorrhage. Before he constructed this bandage, he was in the constant habit of employing a small table-napkin. But the bandage in question is more extensive in its application, while it is extremely simple; as it is applied at the very commencement of labour, and follows it through all its different stages.

N.B. The bandage is made by a poor deformed young woman, (Miss Grierson, No. 6, Bedford Place, Deptford Lower Road) and costs but four shillings. It will last a female for life.

P.S. Mr. G. forgot to mention that, before the bandage is put on, after parturition, its utility would be increased, if a small pillow were placed on the abdomen previously to drawing the tapes.

---

### 35. IPECACUAN EMETICS IN MÆNORRHAGIA.

Dr. Osborne (Assistant Physician to Sir Patrick Dun's Hospital) has published a short paper on this subject in the recent volume of the Transactions of the Association. Although Denman hints at the exhibition of nauseating doses of ipecacuan, in hæmorrhage after abortion; yet M. Caffin appears to have been the first who describes cases in which emetics of ipecacuan were followed by the immediate cessation of the discharge. In the Institution for the Suppression of Mendicity in Dublin, in which there are, on an average, above 1200 women, there is a Dispensary, but no Hospital. Mænorrhage is of frequent occurrence among these paupers.

"I began the use of Ipecacuan by ordering a scruple to be taken as an emetic at night, and I generally directed an acidulous saline purgative to be administered the following morning. The effect produced exceeded my most sanguine expectations. The discharge either ceased within twenty-four hours, or was so much diminished that no more remedies were necessary to insure its entire removal. In some few cases it recurred within a short time, but when this did happen, it was only necessary to repeat the emetic once or twice in order to produce a permanent effect. I met with a few individuals in whom the discharge continued with little alteration after the first emetic, but with these I had only to repeat the remedy on the following night; and in one case alone three emetics were taken before the desired effect was produced."

Five cases, from the books of Sir P. Dun's Hospital, are related merely by way of illustration and authentic proof of the practice.

---

### 36. Cases of Erysipelas Phlegmonodes.

[Mr.Plymsoll—GlasgowRoyal Infirmary.]

*Case* 1. George Ross, æt. 33, hinge-maker, was admitted into the Infirmary, October 31st, 1827, under the care of Dr. Anderson. The skin has sloughed from right arm through its whole extent from shoulders to extremities of fingers, with the exception of a stripe about an inch in breadth on its anterior and inner surface, and a small portion on internal surface of wrist and fore-arm. The raw surface looks clean, and the granulations are red and healthy. On the back of the hand the tendons are exposed, but are becoming covered with granulations. The fingers are in the bent position. Five weeks ago, without evident cause, he observed some redness and swelling over back of hand, which gradually extended up arm. He was delirious for some time —arm was at first poulticed, and about eight days after its commencement, a puncture was made about middle of arm and a large quantity of matter evacuated —it was afterwards covered with flour—has been taking from six to eight ounces of wine daily for the last three weeks—health impaired, but he has been improving within the last eight days—pulse 100—tongue whitish—bowels regular—perspires during the night—has slight cough—ordered to have eight ounces of wine daily—the arm was enveloped in cotton. 11*th*, Cotton to-day removed—granulations healthy, and some formation of new skin at edges—to have bark. 14*th*, Has had a feverish attack with acute pain of right side—pulse 120, full—skin moist—tongue furred—dressings to be removed and a poultice applied—bled to 25 ounces—a blister was afterwards applied to the affected side. 15*th*, Pain of side relieved—bowels free and skin moist, from salts and antimonial mixture. 16*th*, Has had a good night, but pulse to-day very rapid, and pain has removed to the left side, though not acute—tongue dry and furred—one loose stool last night —respiration easy, and he expectorates copiously—sore discharges healthy pus. 17*th*, Has had another easy night, and complains of little pain even on full respiration but pulse not improved, being still rapid and rather weaker—countenance more sunk and sore does not discharge so freely—ægophony is distinctly perceived on right side of chest posteriorly—digitalis was exhibited for some time, but without effect—hiccup supervened—dyspnœa and general prostration increased, and he gradually sunk from this period—he died on the 29*th*. Inspection. Right pleura bore strong marks of previous inflammation—its folds adhered at the anterior superior parts—posteriorly they were covered by small membranes, and contained between them about six ounces of sero-purulent fluid. The part of the lung corresponding to the situation of the effusion was hepatized, and presented an appearance exactly resembling granite—almost the whole of the superior, and middle lobes was in this state—left lung natural in appearance, excepting that there was some coagulable lymph round the larger vessels forming the root of the lung—other viscera sound.

*Case* 2*nd*. Thomas Jack, æt. 42, sergeant of police, was admitted December 1st, 1827, under the care of Dr. Cooper. Left leg and foot are greatly swollen, and of a dull red colour somewhat tense at inferior part of inside of calf, and about foot and lowest third of leg has a boggy feel on pressure—a patch of integuments along outside of dorsum of foot and around outer ancle 10 inches long and four broad, have sloughed, leaving a sore covered by dead cellular membrane of a yellowish colour, and edges are extensively undermined—on back of leg there are several broken vesicles discharging a serous fluid—limb is the seat of severe burning pain—has been confined to bed since disease began and strength is much reduced—pulse 120 small—tongue brown and arid—skin warm and dry—thirst. Bowels open from medicine—some delirium at night. About ten days since fell down a few steps of stairs, and thinks he sprained tarsus—severe pain succeeded on dorsum of foot and the day following he had frequent rigors, and some redness appeared on foot—leeches were applied, but symptoms increased rapidly, and integuments on dorsum of foot which had vesicated, sloughed six days ago—applied emollient and fomenting poultices—and three days since three superficial scarifications were made along lower half of leg. An incision three inches long is now made through the common integuments on inside of calf of leg—sores and wounds dressed with lint dipped in ol. camph: and a bandage lightly applied from toes to knee, which is to be kept moist by a lotion of lime-water and spirits—to have at night 40 drops of laudanum with half a drach of antimonial wine—to have four ounces of wine daily. *2nd*, Slept well last night—pulse of better strength—says he feels easier than for several days past—cont. al. *4th*, Continues to improve—tongue cleaning—slough on foot separating, with free discharge of healthy pus—swelling of leg much diminished. *8th*, The whole slough separated, leaving a tolerably healthy sore—inflammation of leg nearly gone—appetite good—pulse 72. *12th*, Continues to improve. *14th*, Bowels have been slightly disordered since last report, for which he has had calomel and an anodyne enema. *16th*, Bowels regular—sore to be strapped and bandaged—cotton has been since applied—sore cicatrizing fast—health good.

*Observations.*

The medical profession are at present much divided with regard to the treatment of erysipelas phlegmonodes, some practitioners have recommended the practice of making incisions through the affected structures, together with a strict observance of the antiphlogistic treatment, whilst others have reprobated this practice and recommended another, which is diametrically opposite; the exhibition of tonics and stimulants, and have substituted for incisions, the application of leeches and poultices. These different opinions have been equally supported by the highest authorities, and, constituting the leading controversy of the day, have excited an extraordinary degree of interest. The method of treatment which has been adopted in this Infirmary, is a modification of both. Incisions have been practised at every stage of the disease. At the commencement of the disease, mercurial purgatives and antimonials have been administered but when the disease has become fully developed it is characterized by extreme depression, and consequently the exhibition of tonics and stimulants has constituted the general treatment. The practice of making incisions in erysipelas phlegmonodes has been established in this infirmary for the last four or five years, and has invariably proved successful—long incisions are generally preferred. I have had an opportunity of late, however, of witnessing the comparative effects of both methods of treatment (the *practice* and *omission* of incisions) in the cases which have been reported above. I have, therefore, considered it incumbent on me to communicate the result of my observations. These cases are obviously of great importance, as it is only by a comparative observation of the different methods of treatment, that we can determine which is the most advantageous. In reviewing these cases, the evidence is decidedly in favour of incisions. The case of Jack is decisive of this practice. Jack was admitted with symptoms indicating a high degree of irritative fever. An incision was made into the affected limb, through the skin and cellular membrane, immediate relief was experienced—the constitutional symptoms were all immediately mitigated, and he recovered with great rapidity. The cure in this case was unquestionably to be attributed to the incision, as there were no accessory measures employed at the same time that could influence the result, and consequently the efficacy of the measure could not be involved in any doubt. The case of Ross when contrasted with that of Jack, strongly enforces the necessity of this practice. The measures which were employed in this case, were altogether inadequate to arrest the progress of the disease—the disease advanced with great rapidity—the integuments of the arm sloughed—extensive suppuration succeeded—and he died ultimately of inflammation of the lungs. Incisions were of course quite out of the question when this man was admitted into the Infirmary, the whole of the integuments having previously sloughed away. I am convinced that this man's life would have been saved, had incisions been made at an early period of the disease.

Inflammations of the lungs and investing membranes are almost invariably consequent on large surgical operations and extensive wounds. These affections are exceedingly insidious, and the ordinary symptoms so fallacious, that they are completely overlooked, until either from the disease having extended so far, or the exhausted state of the patient forbidding active measures, they become quite uncontrollable, and terminate fatally. I am convinced that stethoscopic examinations would have the effect of obviating, either in a great measure or altogether, these fatal terminations—and I consider it highly incumbent on those gentlemen whose prerogative it is to officiate as hospital surgeons, and have such excellent opportunities for acquiring a thorough knowledge of stethoscopic indications, to make use of the stethoscope in such cases.

This man having died of inflammation of the lungs therefore, does not militate against the inference which has been drawn from this case, as it was obviously a disease consequent on the extensive suppuration from the affected arm. It may be questioned, in a critical examination of this case, whether the local disease might not have been arrested by the application of leeches to the affected part, and whether the event of this case might not have been different had the general abstraction of blood been persevered in, as there was a decided mitigation of symptoms after the first bleeding. I have no doubt of the efficacy of leeches as auxiliaries. I am convinced, however, by the evidence of analogy, that they are incapable (excepting in very mild cases) of subduing the disease. This has been exemplified in the case of Jack—this case also shews the inefficiency of mere scarifications, when both the skin and cellular membrane are involved in the disease—with regard to bleeding being persisted in, the exhaustion in this case was so great as to render it impracticable. Incisions operate beneficially by relieving tension and the local abstraction of blood, chiefly, however, by relieving tension. We should not, therefore, be deterred from making incisions in cachectic or debilitated individuals from a fear of producing an augmented state of debility, by the loss of blood occasioned by an incision; the bleeding may be in a great measure suppressed, (whilst the principal object, the relief of tension, is attained) or advantageously compensated for, by the exhibition of bark and wine, as these measures are not incompatible with each other.

---

### 37. Mania from Sobriety.

There is danger in all sudden changes of long-continued habits, even from intemperance to sobriety, as the following fact proves.

A man who was much given to drunkenness, committed a theft, and was cast into prison, where, of course, he had low diet, and only water to drink. After the first week of his incarceration, he began to evince some symptoms of disturbed intellect, after which he quickly lost flesh, became sleepless, debilitated, and ultimately delirious. In short, mental derangement was unequivocally manifested, and he was tormented with horrible images before his eyes. Dr. Hansbraudt, after examining into the complaint, and the previous habits of the individual, ordered him a moderate quantity of brandy twice daily. The good effects were soon evident. The cerebral disturbance subsided, and he regained his flesh and strength.—*Rust's Magazine, T.* 21.

The above is a species of delirium tremens not uncommon among those who make sudden changes from full living and stimulating drinks to low diet and watery beverages.

38. On the Use of Nitrate of Silver in certain Affections of the Eye. By Isaac Ryall, Esq. Surgeon-Oculist to the National Institution, &c.

[Dublin Transactions, Vol. V.]

In a former number of this Journal, we introduced the substance of a paper by the same Author, on the purulent ophthalmia of infants; and as we know Mr. Ryall to be a purely practical man, who has had wide experience in ophthalmic surgery, we have much pleasure in again introducing him on the tapis.

The nitrate of silver has been found so beneficial in many diseases of the eye and its appendages, that, as might have been expected, its application has become empirical and indiscriminate. It is, therefore, desirable that we should know, from scientific experience, what are those conditions of the organ to which this remedy is peculiarly applicable.

1. *Ulcer of the Cornea.*—This is a very common termination of ophthalmia—a complaint remarkably prevalent among the poor in Dublin, where it assumes a worse character than in any other part of the United Kingdom.

"The cornea, after protracted, and sometimes after comparatively mild inflammation of the conjunctiva, is frequently occupied by an infinite number of small superficial ulcers, appearing as mere depressions or losses of substance in the investing tunic. These, though so minute as to require the closest inspection, and even the aid of glasses for their detection, yet are productive of no small share of irritation and inconvenience. Hence this irritation is sometimes ascribed to primary inflammation, and ineffectual, if not injurious, means of relief resorted to. I have not unfrequently known patients of weakly strumous habits to have been condemned to long confinement in darkened apartments, to a strict antiphlogistic regimen, and even to the influence of mercury, whose miseries might have been, in a great measure, curtailed, had the precise nature of their complaint been timely discovered, and the nitrate of silver applied."

For the superficial ulcer, a solution, containing two grains of the nitrate to the ounce of distilled water, generally answers—if the ulcers are obstinate, the solution may be made stronger, and conveyed to the parts by way of injection. When the ulcers deeply penetrate the laminæ of the cornea, the remedy is still more imperiously demanded, else the organ will be destroyed. In addition to the solution, the caustic itself, or a saturated solution should be applied to the ulcers. A little oil of sweet almonds ought to be dropped into the eye after the nitrate is used. This process is to be repeated on each separation of the eschar, and continued till the ulcer assumes a more healthy aspect, and becomes less painful. The escarotic is then to be laid aside, and the weak solution applied. Mercury has been considered as great a specific in this complaint as in syphilis; but the habits and constitutions of those who are generally the subjects of the former, render mercury, to any extent, inadmissible.

"There is, however, a contra-indication of this remedy under the circumstances of redundancy of lymph, or of interlaminary ulceration or effusion, when the object is, by means of local blood-letting, purgatives, and emollient fomentations, to diminish the vascular action of the part. If considerable pain and inflammation be present, the nitrate of silver must either be accompanied by, or give place to, according to the violence of these symptoms, the means just mentioned; and, if no constitutional taint forbid, a pill composed of calomel, antimony, and opium, should be administered twice a day, until the mouth be slightly affected, or the symptoms recede. It has been the practice in ulceration of the cornea to drop into the eye a little of the vinous tincture of opium twice or thrice a day; but this is known greatly to aggravate the irritation, without in any degree contributing to produce healthy action of the parts."

If no constitutional symptoms forbid, some calomel, antimony, and opium should be administered twice a day, until the mouth becomes slightly affected. The introduction of vinum opii is reprobated by our author.

2. *Pustules.*—These appear in the form of one or more little eminences, either on the sclerotica or cornea—but most frequently at the junction of these two tunics, where, as they advance to suppuration, they bear no small resemblance to little pearls set round the edges of the cornea. This form of ophthalmia is the concomitant of a scrofulous taint. Where the inflammation runs high, leeches and purgatives should be employed ; but the ulcerative process of the pustules will be best restrained by a few light touches of the stronger solution of the nitrate.

3. *Slough of the Cornea.*—As an external remedy for this dangerous affection, " there is none so grateful, or withal so efficacious for throwing off the morbid parts, as a solution of the nitrate of silver, in the proportion of eight grains to the ounce of water, briskly injected against the sloughs, three or four times a day."

" But to obviate and arrest the progress of this serious termination of disease, we must rest our chief reliance on the extract of cinchona, so justly praised by the late Mr. Saunders. Opportunities are afforded to me but too frequently of giving this medicine a fair and full trial, in the cases of young infants born for the most part in the Lying-in-Hospital, and I assert that, greatly as I had been prepossessed in its favour, it far exceeded my expectation, since both shape and function were preserved to an extent I dared not to hope for, in several instances where death of the exterior laminæ of the cornea, or of a segment of its entire thickness, had already taken place, and when the eye had presented the appearance of a disorganized mass."

4. *Protrusion of the Iris.*—" In this case, the nitrate of silver is our chief, perhaps our only resource." The aqueous humour continues to be distilled through the aperture as fast as it is secreted, until the breach is filled up. The effects produced by a few applications of the pencil-pointed nitrate of silver, or the strong solution, are truly gratifying. Healthy action and new growth of parts are thereby quickly obtained—the cornea resumes its convexity—the protruded iris is retracted, and, unless the breach has taken place in the centre of the cornea, vision is completely restored.

5. *Nebulous Cornea.*—This, the result of chronic inflammation, consists in a thickening and increased vascularity of the conjunctiva, and effusion between this membrane and the exterior lamina of the cornea, of albuminous fluid.

" The application of leeches to the inner surface of the lower palpebra, the exhibition of purgatives, and precaution against strong liquors and cold, are the first objects to be attended to. If granulations exist on the palpebræ, they are to be removed by the means hereafter to be mentioned under the head of " granular palpebræ." After the vessels shall have been well emptied by the daily application of two leeches to the conjunctiva of the lower eyelid, and a few purgatives have been administered, a circle, or as large a segment of one as will include the opake portion of the cornea, is to be described with the pencil-pointed caustic on the sclerotic at about two lines distance from its junction with the cornea. After the eschar has sloughed off, ulceration is to be kept up for some time by the same means, care being taken to subdue any excessive inflammation which may be thus produced. The solution of the nitrate of silver, varying according to circumstances in the proportion of from two to six grains to the ounce, is to be frequently injected into the eye, and the ointment of the red oxyde of mercury applied every night to the tarsi, a portion of which may be allowed to be diffused over the surface of the eye-ball."

6. *Albugo.*—" In no case has the nitrate of silver been more abused than in the early stage of albugo, which consists in a deposition of coagulable lymph by the arteries, during the presence of ophthalmia, between the laminæ of the cornea."

" Absorption in this stage is more judiciously attempted by topical blood-letting, and alterative doses of calomel, antimony, and opium, than by local stimulants, whose premature use would be productive of increased vascular action and deposition. When, however, the former have been persisted in for some length of time, which the experience and observation of the surgeon can only determine, it will become necessary to have recourse to the latter, which should consist in a solution of from four to eight grains of the nitrate of silver to one ounce of distilled water, and in a weak ointment of the red oxyde of mercury ; the former to be applied twice or thrice a day, and the latter every night with a camel-hair pencil to the speck."

7. *Leucoma.*—That dense, pearl-coloured, inveterate opacity occupying a part or whole of the cornea, cannot be removed by any means with which we are acquainted.

8. *Staphyloma.*—Mr. R. affirms that, in many instances of those recent staphylomata of young children succeeding to small-pox and purulent ophthalmia, "the growth has been checked, and even the removal accomplished, by producing upon them, and keeping up, for a considerable length of time, artificial ulcers by means of the nitrate of silver."

9. *Granular Palpebræ.*—This is the consequence of long-continued inflammation, especially of the purulent form, producing irritation (by friction) and even opacity.

"The granulations may be shaven off with a scalpel or the shoulder of a lancet, and the abraded surface touched with a strong solution of the nitrate of silver; or, without any previous excision, the caustic may be applied, and repeated after each sloughing of the eschar, until the granulations have disappeared, care being taken, after each of these applications, to wipe the parts with a sponge, and to drop into the eye some oil of sweet almonds."

10. *Ectropion.*—This morbid thickening of the conjunctiva may sometimes be removed by the nitrate—but when the surface is callous, it must be removed by the knife or scissors.

Finally, when it is necessary to remove encanthis, pterygium, and other fleshy excrescences by the knife, the subsequent aid of the nitrate of silver will be required, for their ultimate destruction, and for the encouragement of healthy granulations.

---

39. ARTIFICIAL ANUS IN THE VAGINA.

To cure the above-mentioned loathsome disease, an operation, equally desperate and unfortunate, has been recently performed by M. Roux, at LA CHARITÉ. Had it happened on this side of the Channel, in the hands of one of the ELECT, we should have heard nothing of it:—But had a Cooper, a Travers, a Brodie, a Bell, a Keate, or, in short, any one, *not of the coterie*, performed the operation, the TOCSIN would have been sounded in every town and village between Dover and Stromness.

A young woman, after a hard labour, was found to discharge the fæces through the vagina. She was admitted into LA CHARITÉ under M. Roux, who discovered the artificial anus behind and towards the left side of the cervix uteri. It was proved that the perforation was not in the rectum or colon, for large injections, pushed up into the latter, came away per anum, and none by the artificial passage. It was, therefore, naturally conceived, that some portion of the ileum had descended there, and got nipped by the pressure of the child's head in parturition. The poor woman found her existence so wretched, under these circumstances, that she implored for an operation, no matter how hazardous, if it promised any chance of cure. All other means failing, M. Roux determined to open the abdomen—search for the injured intestine—separate it from its adhesions—cut it across, if it were not already divided—unite the two ends by suture—and leave the rest to the efforts of Nature. The boldness of the attempt deserved a better fate; and although a great and fatal mistake was made in the operation, Heaven forbid that we should imitate the bad men of our own country, by calling down imprecations on the head of that surgeon, who hazarded his reputation to alleviate the miseries of a fellow-creature!

We shall not detail the steps of this proceeding. An opening was made into the abdomen between the umbilicus and pubes—the intestine was searched for, and supposed to be found—the two ends were drawn out, incised, and reunited by suture—and then returned into the abdomen. The patient died in 38 hours afterwards. The account of the dissection, as well as that of the operation, defies comprehension in many of its parts. We can gather from it, however, that the lower portion of the ileum had been joined to the sigmoid flexure of the colon—and, consequently, that two portions of intestine were left opening into the general cavity of the abdomen—the lower extremity of the colon, and also the lower extremity of the ileum. It is difficult to conceive how this could have been done—but knowing, as we do, the anatomical knowledge, and manual dexterity, of M. Roux, it would probably have been much more difficult to have done otherwise than he did, under the existing circumstances of the case. This, we conceive, is the fair, the just, the professional inference to be drawn, when a surgeon of acknowledged ability, education, and experience, has fallen into an error.

It appears, from the account given in our cotemporary, the Medical Gazette, that much discussion has arisen in the Parisian saloons respecting this operation—some applauding, and others censuring it. That no illiberal criticism, no malicious misrepresentation of the operation, will appear in the Parisian journals, we are quite positive; and sorry are we to observe, in the English reporter of the case, a tincture of that censorious spirit which it is the duty, as well as the wish, of the Medical Gazette to expose and stigmatise in the present juncture.

"We record the case, not as one to be imitated, but shunned; and as a most *unwarrantable* attempt, on the part of the surgeon, *who appears to have set the desperate chance—the bare possibility of success*, and consequent renown of himself, *against the almost incalculable hazard to his patient.*"

The reporter first informs us, that—"this disgusting malady embittered her life to such a degree, that she *continually* implored to be relieved by an operation, *however dangerous and painful that might be.*" Then again, after the patient had lived, "for a long time," on a small quantity of rice broth twice in the 24 hours, without any relief—" the entreaties of the woman became *every day more pressing*, and M. Roux, anxious to relieve her if possible, first planned the operation, &c." How the reporter could, after putting the above on the record, proceed to accuse the Parisian surgeon of wantonly hazarding the life of his patient for the sake of gaining "renown to himself," we leave him to explain. In this instance, he has put himself too much on a par with the Borough reporter, whose conduct has excited such universal disgust.

### 40. Neuralgia; or Tic Douloureux.

The paper lately read by Sir Henry Halford at the Royal College of Physicians, before a very great concourse of the most eminent men in the profession, has excited considerable interest, in consequence of the attempt made by the talented author of the paper, to point out one of the occult causes of this terrible disease—namely, lesion of bone. Sir Henry very properly stated, that in many, indeed in the majority, of neuralgic affections, the pain was excited sympathetically—and, consequently, the source of the malady was not where the effects were felt. It was, however, to the disease, as it exhibited itself in its most awful forms, and in some of the branches of the fifth pair of nerves, that he principally adverted, and that with the view of shewing, by some cases detailed, that the neuralgia was caused by diseased bone. In one of these cases, there was an exostosis of the alveolar process—in another, there was disease of the antrum highmoreanum—and in a third, and the most remarkable of all, there was a prodigious deposit on the internal surface of the skull, (which was exhibited at the College) like frost-work, or rather like petrifactions, which must have caused great pressure on the brain—was probably the cause of the neuralgia—and ultimately ended, as many of these cases end, in apoplexy. This was the termination of Dr. Pemberton's life. The skull was not accurately examined; but there was great thickening of the os frontis—and a bony deposit in the falciform process of the dura mater. It is a curious fact, recorded by Sir Henry Halford, that Dr. Pemberton, previously to the development of the neuralgia of which he died, had an abscess twice in the frontal sinuses. There is little doubt, therefore, that his case was one of those dependent on disease of bone.

The experienced and modest author (for modesty generally accompanies experience, and *vice versâ*) did not attempt to account for all cases of tic douloureux on this pathological principle; but merely related these cases, in order to draw the attention of the profession to a cause not generally suspected, and, consequently, to elicit further investigation. As, in the course of the present, or, at all events, of the next number, of this Journal, we shall have occasion to introduce a very extended article on neuralgia, we will not here anticipate any portion of the information which we shall soon lay before the public. We may, however, mention, that Dessault found a diseased state of the bony foramen, through which a branch of the third pair, affected with neuralgia, passed—and that the late Mr. Wilson maintained in his lectures, that disease of bone was not an uncommon cause of tic douloureux. Many cases are on record, (one, for example, by Mr. Swan) where accidental injuries have been followed by the painful affection in question. Indeed, a physician of this metropolis, more celebrated for his convivial talents, than for his pathological acumen—gained great credit once, for detecting the cause of tic douloureux in a gentleman of distinction. This gentleman was a great equestrian—became affected with neuralgia—and consulted Dr. B———. The knowing M.D. after hearing the detail of sufferings, asked, as a first question—or rather affirmed, point blank, that the patient had had a fall from his horse. No great exertion of memory was necessary, on the part of the equestrian patient, to corroborate the sapient observation of the Doctor, who was thenceforth (for a time, at least) the MAGNUS APOLLO of the fashionable world!*

That the terrible disease under consideration is produced by *many causes*, we shall shew, by abundant proofs, in an approaching article—and, among other things, we shall bring forward strong corroboration of the view taken by Sir Henry Halford—as *one* cause, not *the* cause, of neuralgia.

---

41. REVIEW OF SOME OF THE SURGICAL CASES WHICH HAVE LATELY OCCURRED IN THE ROYAL INFIRMARY OF EDINBURGH—A CLINICAL LECTURE DELIVERED BY DR. GEORGE BALLINGALL, on the 28th February, 1828. 4to. pp. 28. Edinburgh, March, 1828.

We are extremely glad to see Dr. Ballingall set so good an example to the surgeons of Edinburgh—and indeed to the surgeons and physicians of all public institutions. The publication of clinical lectures would be productive of infinite benefit, as they would convey the most authentic information of which medical literature is susceptible. In these reviews or retrospects of cases, the clinical lecturer appeals at once to the testimony of his auditor's senses, all or most of which auditors have been eye-witnesses of the cases forming the subject of comment. We shall endeavour to diffuse the contents of this lecture as widely as possible.

1. *Injuries of the Head.* It is to this important subject Dr. B. first draws the attention of his pupils. The case of James Wylie, aged 28, is reviewed. He was found lying at the foot of a precipice, over which he was supposed to have fallen in a state of intoxication, and was brought to the hospital in a condition of stupor and insensibility, from which he was recovered by local and topical blood-letting. The case was a fine specimen of *concussion*.

The cranial *fractures* appear to have all been of a formidable character. The following is a very remarkable case.

"This man, apparently about fifty years of age, was brought in by the police, having been found in an area into which he had fallen; he was evidently in some degree under the influence of spirits, of which he smelt strongly, but at the same time shewed symptoms of a fatal injury at the base of the skull; hæmorrhage from the ears, continued delirium, and effusion under the posterior part of the scalp; the appearances altogether so unfavourable, that you may remember my having remarked at the visit that he was scarcely a subject of surgical practice.

"He survived the accident for two days, his delirium continuing unabated so as to require the coercion of a strait-jacket until within a few hours of his death."

Dissection disclosed a dreadful amount of injury. Blood was plentifully effused beneath the scalp. One fracture extended upwards from the posterior and inferior angle of the left parietal bone, a portion of which was depressed. Another fracture nearly surrounded the superior occipital fossa of this side. A third passed through the petrous portion of the temporal bone, the greater and lesser alæ of the sphenoid, and, in the posterior part of the ethmoid, united with nearly similar fractures of the opposite side.

---

* An exfoliation of bone from the nose, where the injury had been sustained in the fall, was followed by relief of the TIC. This completed the Doctor's fame.

"Between the skull and dura mater of the superior occipital fossa a large coagulum of from two to three ounces of blood was collected and adhered strongly to the membrane, the corresponding part of the brain being much flattened. Underneath the pia mater of the superior part of the right anterior and middle lobes, blood was extravasated in several places. The lower surface of the left middle lobe appeared to have been lacerated, and from this point a cavity containing about two ounces of blood, mixed with softened cerebral substance extended into the posterior lobe, the parietes of this cavity were pulpy, and of a reddish grey colour. The inferior surface of the opposite lobe had undergone a similar change but to a less degree." It is not a little surprising that the usual symptoms of *compression* were here nearly wanting, while those of *excitement* were prominent till the last.

Another remarkable case of compound fracture of the skull is detailed. The patient was irrational and violent—the wound was in the forehead—and a portion of depressed bone having been elevated, and a piece of brain having escaped —"the patient instantly recovered his senses, and answered questions rationally." He soon relapsed, however, and died in 48 hours from the receipt of the injury. On dissection, it was found that fractures had run down through the orbital plates to the base of the skull, with laceration of the dura mater, brain, &c.

"As young men, who have necessarily much to read and much to learn, it may be well to caution you here against the extremes into which surgeons have run in estimating the advantages of artificial interference in injuries of the head. 'Look,' says Mr. John Bell, 'into the books of the ancients, and you would believe that every capillary fissure was attended with peculiar danger, and that without the most adventurous operations the patient could not live; name me,' says he, 'one absurd or cruel measure—the amputation of large pieces of the scalp—the widening of fissures—the perforating the cranium with many trepans—and opening the dura mater for every idle suspicion or imaginary purpose; name me, any extravagance for which their works do not afford us a precedent.' 'Turn again,' says Mr. Bell, 'to the works of more modern authors, and you would be persuaded that the more violent the fracture the less the danger; that your patient, though he lie in a deadly stupor with fractures of the skull, or deep wounds of the brain, needs but to lie undisturbed or unassisted to insure his perfect recovery.'

"Even in very recent times we find the most distinguished surgeons of the day inculcating practices almost diametrically opposite, Mr. Pott, in England, encouraging a use of the trepan almost unlimited, and Desault in France, latterly renouncing it *in toto*,—his practice apparently becoming enfeebled as his experience increased."

II. Traumatic Delirium. In three cases of simple fracture (one of the ribs, and two of the lower extremities) delirium tremens—or, as we would term it—traumatic delirium supervened. The first case related was a female (Mary Kidd) aged 55 years:—

"'The whole of the left upper extremity is ecchymosed and much swollen; there is a distinct crepitus near the upper end of the humerus, and during the rotation of the bone the head remains motionless, the lower end of the bone is drawn in towards the chest, pulse 100 and feeble, belly costive, tongue furred. Injury was the consequence of a fall down some steps on Saturday last; the pain at the upper part of the limb has been constantly upon the increase.'

"On the 4th of January she was observed to be labouring under symptoms of delirium tremens; she had been constantly talking during the preceding night, and did not answer questions rationally, had much tremor of the hands; her pulse 90; skin cool, and tongue moist.

"These symptoms continued with considerable variation, and with occasional intermissions, until about the 18th, when a considerable slough was observed on the sacrum and right buttock. This increased progressively notwithstanding the use of every means to protect the parts from pressure, and she expired on the 28th, having been treated during the progress of her complaint with large and repeated doses of opium, with wine, and with diluted spirits according to circumstances; of the former she took upon one occasion at the rate of 20 grs. in the 24

hours, and her symptoms were more than once apparently removed by this treatment.

"The fractured portion of the bone was exhibited to you at a subsequent lecture, and the fracture was found, as it had been represented, to extend through the anatomical neck of the humerus, passing also obliquely downwards into the shaft of the bone. Although the parts appeared to have been in very accurate apposition, and to have had a supply of nutriment from the investing membrane, sufficient to preserve their own vitality, yet no step towards the reunion of the fracture had taken place."

The second case was that of a man, aged 44 years, who was admitted with an inflamed ankle, the result of a fall received a few days previously. The skin was of a dark red colour, very tense, with some incipient vesications; but with so little displacement of bone that a fracture, at the first glance, was not suspected. When this was discovered, leeches and proper means were used, and every thing appeared to be going on well for ten or eleven days, when "DELIRIUM TREMENS" supervened, and proved fatal in four or five days. The patient exhibited all the symptoms which we observe in the advanced stage of typhus. Dr. Ballingall alludes to the paper on traumatic delirium, by Baron Dupuytren, which we lately published in this Journal; but refers to the writings of physicians for full information on this subject. We cannot admit that this disease is identical with MANIA A POTU, described by the authors in question.

III. TETANUS. This is the case published by SCOTUS, and adverted to at page 170 of this number. SCOTUS has embellished (if such an expression be allowed) the particulars of this melancholy accident; but as the main facts of the case are substantially correct, we can only censure the *animus* with which the minor ones are exaggerated.

IV. HERNIA. The following case of strangulated hernia, with the clinical observations of the operator, we shall give from the report before us.

"This patient, *James Davidson*, about 45 years of age, was brought into the hospital with a hernial tumour in the left groin, stated to have been in a state of strangulation for the five preceding days, during which no evacuation from the bowels had taken place. The taxis, in conjunction with bleeding, and the tobacco injection, had been ineffectually employed previous to my reaching the hospital; and without any farther effort I proceeded to the operation. In consequence of the tender state, and near approach to gangrene in the protruded part of the bowel, I was induced to make the division of the stricture unusually free, so as to permit the gut to be returned without the risk of laceration; the patient's bowels were freely relieved during the course of the ensuing night; but his strength was greatly exhausted, and he sunk on the following morning, having survived the operation only about fourteen hours.

"Although this operation was not attended with the happy result which I have generally experienced, yet it afforded a good illustration of two points in reference to the treatment of this disease, which I am desirous of taking every opportunity to inculcate, the necessity of an early operation, and the safety of its performance. In this case, considering the patient's debilitated state, and the long existence of the strangulation, I was almost induced to regret that any auxiliary means had been attempted. The tobacco injection particularly is a measure, against the indiscriminate use of which I am disposed to caution you. I have seen so few instances of the success of this remedy, and so many examples of its apparently producing noxious effects on the system, that I am inclined to consider the operation a much less hazardous expedient than the tobacco injection in those cases of strangulated hernia, in which it is usually resorted to. You saw, from the preparation which was exhibited to you by Mr. Russell, that, although, in the present case, the incision in the ring was nearly double the extent to which I have usually found it necessary to carry it, yet I ran no risk of wounding the epigastric artery; and this circumstance I am induced to notice, because I apprehend that where we are desirous to encourage the more frequent performance of an operation, we shall be most likely to attain our object, by showing that, if not always successful, it is at least generally safe."

V. *Tumours and Excrescences.* The period embraced by Dr. Ballingall's report appears to have been singularly fertile in bringing forward a number of excrescences and tumours in different parts of the body. The following is a curious and interesting sample.

"Another excrescence of an anomalous character presented itself in the person of Mary Goodfellow, aged 16, who was admitted on the 6th of December, with a cuticle in various parts of the body presenting the appearance of old superficial cicatrices, apparently the result of some general cutaneous eruption; at the left commissure of the lips, at the anterior margin of the left axilla, and on the left fore-arm, immediately below the flexure of the elbow-joint, were prominent warty excrescences; and in the angle between the right labium pudendi, and top of the corresponding thigh was another excrescence of the same character, nearly as large as a duck's egg, it was of a soft warty texture, its surface apparently consisting of numerous granular bodies, of a florid red colour; and was by many very aptly compared in its appearance to the roe of a salmon. The history given of the origin and progress of the complaint, by the patient and her mother, was exceedingly unsatisfactory, and in many respects altogether contradictory; on one occasion it was stated to have been growing from her infancy, and on another to have originated only a few months ago; by some it was considered as a form of frambœsia or yaws, by others as a case of sibbens, and by others as a venereal affection; my own opinion, at first, was rather in favour of the latter supposition, chiefly from an apparent desire on the part of the patient and her mother to conceal its true origin, and from its resembling in appearance, those cauliflower excrescences, frequently met with on the prepuce and glans of the male, as a sequela of venereal ulcers or abrasion; at all events, the disease was obviously of an extended and constitutional character, and hence, upon consultation with my colleagues, it was agreed to try the effects of constitutional treatment.

"The girl was therefore put upon a course of mercurial pills, and a solution of corrosive sublimate, directed as a local application to the excrescence in the groin, the one in the axilla having been previously removed by a scalpel."

On the 13th Dec. we find the mouth sore—the swelling in the groin much increased—and a few of the largest of the small granular bodies composing the bulk of the tumour sloughing and falling off daily. On the 5th Jan., the tumour was removed by excision, and some smart fever followed; but the excitement having subsided, the wound began to heal kindly, and soon cicatrized. Her general health improved, and she was subjected to no farther medical or surgical discipline.

We shall be able to introduce but one more case from this report, in the present review.

"On the 11th of January, Elizabeth Hay was admitted for the purpose of having a tumour removed from the scalp, which is thus described in the journal:—'Over the vertex of the head, and attached by a broad base, is a large firm tumour rather greater than a clenched fist. It is moveable on the skull, and in some parts has burst, discharging a thick yellow matter; around the places where it has burst, it is of a soft consistence, towards the base it feels harder. All over the body are small soft tumours, generally attached by soft pedicles, and from the size of a pea to that of a walnut.

"'States that her skin has been, from her infancy, covered with these tumours, which gave her no inconvenience till within the last three years, when the one on the vertex became painful, swelled, and attained its present size. Three weeks ago it burst, and has since continued to discharge pus. General health good, bowels regular.'

"This tumour was removed by Dr. Hunter, and on investigating its structure, you saw that whatever might have been its original nature, whether akin to the other tumours with which this patient's body was studded over, or not, it had, previous to its removal, assumed a carcinomatous character; you saw at some points the appearance of fibrous bands passing through it in different directions, with matter of a dirty yellowish colour, and atheromatous consistence occupying the interstices between them; at other points the texture of the tumour was completely broken down, and it discharged a most offensive ichorous matter, in so much as to be loathsome to the poor woman, who earnestly entreated its removal.

"In doing so, it was found that the tendon of the occipito-frontalis muscle was involved in the structure of the tumour and the pericranium was left bare after the operation. The sore healed kindly and the patient was dismissed cured on the 20th of February."

The other tumours on this woman's body did not come under surgical treatment. They constituted a very rare disease, termed molluscum pendulum, by Dr. Bateman. Some remarkable instances of tumours within the orbit are next detailed; but these we shall notice separately in another part of our Periscope.

Dr. B. concludes his clinical lecture with a fervent aspiration that hospital practice may be made more available to the profession, by the publication of clinical reports—"documents of more importance than volumes of idle, uninteresting, or partial detail." Dr. B. is far from undervaluing individual cases, "when recorded in the candid, unaffected language of a Pott or a Hey"—but as a large majority of the cases furnished by individuals "exhibit only the bright side of the picture, they become unsafe guides for regulating our future practice." Well may Dr. B. observe that—"it is not in the occasional detail of an isolated case, whether published on the one hand as a PUFF, or, on the other, as a SATIRE, that the improvement of our profession is to be sought."—So say we!

---

### 42. INSANITY.

An interesting discussion has recently taken place, in the Westminster Society, on a paper by Dr. Epps, on the subject of *insanity*. The essayist began by remarking the importance of a knowledge, "*wherein insanity consists*," to the public at large, but more particularly to the medical man; inasmuch as, to the latter, is committed the important trust of consigning a fellow creature to confinement among the insane;—a trust, perhaps, the most responsible that can be conceived. Dr. E. then pointed out a circumstance, or rather doctrine, which had tended very much to impede the progress of the knowledge and the treatment of insanity. This was no other than the doctrine of its *incurability*; a doctrine which Dr. E. conceived to depend upon the metaphysical notion, that *the mind exists unconnected with matter*; and being thus unconnected, means acting *organically* could have no effect, and, therefore, such means were seldom used.

The object of his remarks, Dr. E. stated, was to prove that insanity is an *organic* (corporeal) disease; and, in establishing this view, Dr. E. treated upon the following points,—a definition of insanity, and the terms referring to its kinds—some general probabilities—erotic monomania, and its pathology—and some notices on the best treatment of insanity generally, and the theory of that treatment.

On the first point, it was stated that the mind might be advantageously considered as divided into *affective* and *intellectual* faculties, inasmuch as sometimes one class was affected with disease, sometimes the other, sometimes both. Dr. E. then gave cases, wherein the affective faculties only were affected; cases in which the intellectual only; and cases in which both were affected. Insanity was then defined to be, a disease of one or more faculties of the mind, of the manifestations of which the patient is not conscious, or, if conscious, is not able to control. Insanity was considered as the *genus*, and as *species*, the three kinds already noticed and illustrated. As varieties of these, *monomania* and *polymania* were mentioned; monomania being applied to those cases where only *one* of either class of faculties was affected;—polymania, when *more than one*.

Idiotism was defined to be a *connate deficiency* in any one or more of the faculties: *fatuity*, the same *brought on by disease or age*.

The general probabilities in favour of insanity being an organic (or corporeal) disease, were stated to be, first, that insanity is an hereditary disease: second, the period between 30 and 40, in which most people that are insane have become affected, (all the bodily and mental powers being then in their greatest activity): third, bodily changes, such as menstruation, pregnancy, &c. bringing on this malady: fourth, the effects of season and weather upon insane individuals: fifth, the periodical nature of the attacks of insanity: sixth, the cessation of sleep, &c.

After stating these general probabilities, Dr. E. proceeded to bring forward the particular species of monomania, called *erotic*, known, when affecting women, under the name of *nymphomania*; when attacking men, under that of *satyriasis*.

A description of this disease, by Pinel, was given; after which, Dr. E. related 14 cases of erotic monomania, in which the *cerebellum*, particularly that part called the *vermiform process*, was affected with organic lesion; and argued thence, that erotic monomania consists in a *disease of the cerebellum*.

A reference was then made to the successful treatment of the insane at the Retreat, and it was maintained that this success depended upon the excitement of faculties not diseased. By determining action to the organs of these, the part diseased was enabled to regain its healthy activity, and the patient recovered; and Dr. E. concluded, by maintaining that it is impossible to explain the facts connected with insanity, except by the theory, that the brain is the organ of the mind, and that the brain consists of a plurality of organs, some of which may be diseased, and some healthy; the former giving rise to diseased, the latter to healthy, manifestations of the faculties dependent on the organs.

Upon the conclusion of these remarks, Mr. Bennett rose, and stated his objections. He maintained that the author said *mind came from matter*. To this Dr. E. replied that his statement was, that mind was known only as *exhibited through matter*; and that to say that he said that the brain is the mind, because he asserted the mind is exhibited through the brain, is equally absurd, as to assert that, should he say the *will* acted through the *muscles*, it was his opinion that the muscles are the will. Mr. B. then attacked phrenology, which had nothing essentially to do with the subject before the Society; this being that, in a particular species of mania, a particular part of the cerebral mass was affected. The opinion stated, that this particular part was connected with the sexual desire, the diseased activity of which constitutes erotic monomania, gave Mr. B. an opportunity to attack phrenology, which maintains that the brain is a congeries of organs. The objections of Mr. B. on this point are replied to in Dr. Gall's work on the Functions of the Brain; and, therefore, reference need be made only to another circumstance mentioned by Mr. Bennett, viz. that some animals that have the sexual desire have no cerebellum. To this it was replied by Dr. E. that Dr. Gall had dissected the different species of animals of the different classes, and had found that all animals having the sexual desire, have the cerebellum, at least that part called the *processus vermiformis*, which is the primitive portion; that, in the mammiferous, superadded to this primitive part, are the two lobes of the cerebellum; and that, in the oviparous, insects, fishes, amphibia, and birds, the lobes were generally absent, but the vermiform process present.

Some gentleman rose, and mentioned that, in 20 cases of epilepsy, the cerebellum was found diseased. Dr. E. stated that he was inclined to conclude, from a case at present existing, where epilepsy has been brought on by excessive sexual gratification, that these cases might have had some similar occasion.

It was observed by Dr. Ley, that disease of the brain was not always apparent in cases of insanity; in fact, that numberless cases have occurred, where no disease could be traced. To this Dr. E. replied, by stating the experience of Voisin, Haslam, Lawrence, Esquirol, and others, who maintain the opposite, and whose opportunities of making observations cannot be doubted. It was hinted, in addition, that our inability to detect disease does not militate against disease existing. How often does amaurosis exist, without any perceptible change in the organ of vision? No doubt some change does exist, but we have not detected the same.

It was then objected by a member, that the sexual organs might be the cause of the diseased state in erotic monomania, and the cerebellum and its affection might be only a coincidence. To this Dr. E. replied, by mentioning cases of eunuchs, of women who had no uterus, of old men, whose genital organs are in a state of inactivity, who have had the sexual desire; and mentioned the fact, well known to all, that the genital organs are often diseased, without exciting erotic monomania. In addition, Dr. E. referred to several cases, where the testicle being removed on one side, the opposite lobe of the cerebellum diminished in size; to others, where, from wounds on the nucha, the sexual desire was lost; and, finally, to the circumstance, that children of three four, five, and six years of age, had experienced the sexual desire, before the genital organs were developed.

Dr. Ley strenuously maintained that the *mind itself* is affected in insanity, and not *its organs*. Dr. Johnson stated that his observations had led him to conclude that, in cases of insanity, there exists functional or organic disease. Dr. Wright, of the Bethlem, stated that, in 100 cases of insane individuals, whose brains he had examined, 90 shewed evident marks of disease; and the others some signs, such as the bloody points on cutting through the hemispheres. Dr. Burrows had seen one case, where he could discover no evident disorganization; but acknowledged that, in all cases of insanity, there was corporeal disorder.

Several other objections were made, and answered, but as some of them were personalities, and others relating to phrenology, and as Dr. Epps offered to meet any gentleman who would bring forward the latter science, no further notice need at present be taken of them.

No reader of this Journal will accuse us of advocating the doctrine of materialism; but we cannot help entering our protest against the doctrine of *mental disease*. Mental *death* is a necessary consequence of such doctrine. The material organ or organs of the mind must become disordered or diseased, before the *manifestations* of the soul can be pronounced in a state of aberration. But to suppose that the soul, the mind, the sentient principle, or whatever else we may call that which *thinks*, should become *sick* or diseased, except from disorder or disease of the corporeal organ by which it is manifested, is a doctrine befitting the days of monkish ignorance, and totally untenable in the present æra.

For our own parts, we have no hesitation in saying that Dr. Epps did himself infinite honour in the paper presented to the Westminster Society, as well as in the prompt and admirable manner in which he met every argument that was brought against him. We shall be extremely deceived, if Dr. Epps does not turn out to be a distinguished ornament of our profession.

P.S. In the third sitting of the Society, on the subject of monomania, Dr. Epps had a more severe ordeal to go through than on any of the preceding nights. Against him was arrayed a most formidable phalanx. SCIENCE poured forth its proud researches—wit and ridicule left not an arrow in their quivers, all being expended on the field of phrenology, to which the doctor's opponents had artfully shifted their ground—Eloquence, with dulcet accents and melodious cadences, *prolonged* the torrent of declamation—and CANT itself preached forth its little anathema against the *immorality* of making insanity a disorder or disease of the brain, as the organ of mind! But all would not do. Dr. Epps, with irresistible strength, but the meekness of a lamb, laid his numerous opponents, one by one, so softly and gently on their backs, that they scarcely felt the fall! He bravely maintained that the brain is a congeries of organs, each adapted for the *seat* and *manifestation* of some faculty of the mind or soul—and, as a natural consequence, argued that it was not the soul or mind which became diseased in insanity, but merely the material organs through which the faculties of the mind were manifested. We consider this localization of the mental faculties as subversive of materialism —grateful to the Gods, and gratifying to man.

Tu pias lætis *animas* reponis
*Sedibus*, virgâque levem coerces
Aureâ turbam—superis deorum
gratus, et imis.

No doctrine, we conceive, in the whole range of physical or metaphysical inquiry, is so well calculated to support the dreary desolating prospect of annihilation, as that which attributes to the MIND the liability to SICKNESS. No advocate of this doctrine can pretend to maintain the immateriality or the *immortality* of that which is subject to *disease*. If the mind is subject to one, two, or three kinds of disease, as mania, monomania, idiotcy, &c. it is impossible to say that it is not liable to dozens of diseases. It may have *jaundice*, and see through a distorting medium—it may be *bilious*, and then it becomes waspish, fretful, and irritable—it may catch an ague, as did the soul of Cæsar, "when he was in Spain," and then—

"Its coward lips will from their colour fly."

In short, there is no end to the absurdities involved in the doctrine of *mental* diseases. No man upholding this doctrine, can maintain that mind is not matter—and, if matter, it must be brain. Adieu, then, to the exalted sentiments of a Plato, or a Cato, that "the soul, secured in her existence, smiles at the drawn dagger, and defies its point."
